# Frailty assessment in adults undergoing allogeneic hematopoietic cell transplantation: insights from a multicenter GETH-TC study to optimize outcomes and care

**DOI:** 10.3389/fimmu.2024.1512154

**Published:** 2025-01-07

**Authors:** María Queralt Salas, María Teresa Solano, Mónica Baile González, Marina Acera Gómez, Laura Fox, María del Mar Pérez Artigas, Ana Santamaría, María del Carmen Quintela González, Andrés Sánchez Salinas, Joaquina M. Salmerón Camacho, Verónica Illana Álvaro, Zahra Abdallahi-Lefdil, Javier Cornago Navascues, Laura Pardo, Sara Fernández-Luis, Leddy Patricia Vega Suárez, Sara Villar, Patricia Beorlegui-Murillo, Albert Esquirol, Isabel Izquierdo García, Sonia Rodríguez González, Alberto Mussetti, Esperanza Lavilla, Javier Lopez-Marín, Silvia Filaferro, Ángel Cedillo, Leyre Bento, Anna Sureda

**Affiliations:** ^1^ Unidad de Trasplante de Progenitores Hematopoyéticos, Servicio de Hematología, Hospital Clínic de Barcelona, Barcelona, Spain; ^2^ Servicio de Hematología, Complejo Asistencial Universitario de Salamanca/IBSAL, Salamanca, Spain; ^3^ Departamento de Hematología, Hospital Universitari Vall d’Hebron, Barcelona, Spain; ^4^ Departamento de Hematología, Hospital Álvaro Cunqueiro, Vigo, Pontevedra, Spain; ^5^ Unidad de Trasplante de Progenitores Hematopoyéticos, H.C.U. Virgen de la Arrixaca, Murcia, Spain; ^6^ Departamento de Hematología, Hospital Universitario de la Princesa, Madrid, Spain; ^7^ Departamento de Hematología, Hospital Universitario Fundación Jiménez Díaz, Madrid, Spain; ^8^ Unidad de Trasplante de Progenitores Hematopoyéticos, Servicio de Hematología, Hospital Universitario Marqués de Valdecilla, Santander, Spain; ^9^ Departamento de Hematología, Clínica Universidad de Navarra, Pamplona, Spain; ^10^ Servicio de Hematología, Hospital de la Santa Creu i Sant Pau, IIB-Sant Pau and Jose Carreras Leukemia Research Institute, Universitat Autonoma of Barcelona, Barcelona, Spain; ^11^ Departamento de Hematología, Hospital Miguel Servet, Zaragoza, Spain; ^12^ Institut Català d’Oncologia - Hospital Duran i Reynals, IDIBELL, Universitat de Barcelona, Barcelona, Spain; ^13^ Servicio de Hematología, Hospital Universitario Lucus Augusti, Lugo, Spain; ^14^ Hospital Universitario de Alicante, Alicante, Spain; ^15^ Grupo Español de Trasplante de Progenitores Hematopoyéticos y Terapia Celular, Madrid, Spain; ^16^ Departamento de Hematología, Hospital Universitario Son Espases, Palma de Mallorca, Spain

**Keywords:** frailty syndrome, HCT Frailty Scale, allogeneic-HCT, pre-habilitation, survival

## Abstract

**Introduction:**

This multicenter prospective study sponsored by the *Grupo Español de Transplante Hematopoyético y Terapia Celular* (GETH-TC) explores the use of frailty assessments in allo-HCT candidates.

**Methods:**

Frailty was measured using the HCT Frailty Scale at first consultation and HCT admission in 404 adults from 15 HCT programs in Spain. Based on the results, patients were classified into fit, pre-frail and frail categories. Allo-HCT outcomes were analyzed according to the results obtained from the HCT Frailty Scale. Data was collected prospectively and all patients signed informed consent.

**Results:**

At first consultation, 102 (26.2%) patients were classified as fit, 248 (61.4%) as pre-frail, and 50 (12.4%) as frail. During the study, 62 (15.2%) patients participated in a pre-habilitation program. Among non-pre-habilitated patients (n=342), the proportion of fit patients decreased from 26.6% to 16.7%, while frail patients increased from 12.7% to 19.9%. In contrast, pre-habilitated patients (n=62) showed improvements, with fit patients increasing from 24.2% to 46.8%, and frail patients decreasing from 9.7% to 3.2%. Multivariate analysis confirmed lower OS (HR 2.52, P=0.002) and higher NRM (HR 2.69, P=0.013) in frail patients at HCT admission compared to fit ones, with a trend towards lower OS in pre-frail patients (HR 1.54, P=0.097).

**Conclusion:**

This study highlights the feasibility of incorporating the HCT Frailty Scale into clinical practice, confirms its negative impact of frailty on transplant outcomes, and suggests that frailty is dynamic and potentially reversible through pre-transplant interventions.

## Introduction

Allogeneic hematopoietic cell transplantation (allo-HCT) offers a potential cure for patients with high-risk hematologic disorders (1). Advances in the transplant methodology have substantially decreased transplant-related mortality within the first 2 years post-allo-HCT, from 40% in the late 1980s to 10-20% today (2–5). These advances have expanded the pool of patients eligible for allo-HCT, particularly including older adults and those with comorbidities (6, 7). However, despite these improvements, allo-HCT still carries risk of mortality and morbidity, underscoring the importance of meticulous patients’ selection (8–11). The frailty status of patients emerges as relevant information for the advancement in this direction.

While frailty assessment has traditionally been restricted to geriatric populations, recent research has demonstrated the usefulness of information about the frailty status of candidates in allo-HCT settings (12–14). To the advancement of frailty assessment and its use in clinical practice in the allo-HCT setting has contributed the development of frailty scales inspired in those used in geriatric settings, but adapted in a cost-effective way to the transplant ones (15–25). Frailty programs in allo-HCT settings have documented an incidence of frailty among adult candidates that ranges between 8% and 25%; have demonstrated the statistical association between patient`s frailty and the likelihood of transplant complications and mortality (15–25); and start to provide first evidence that frailty is dynamic in allo-HCT patients and eventually reversible through pre habilitation programs.

These trends in frailty research motivated the *Grupo Español de Transplante Hematopoyético y Terapia Celular* (GETH-TC) to initiate this study, aimed at assessing frailty in allo-HCT patients across HCT units in Spain. The study implements the HCT Frailty Scale, developed at Princess Margaret Cancer Center in Toronto, Canada (26, 27), to classify transplant candidates into three frailty levels—frail, pre-frail, and fit—at both the first consultation and HCT admission, in order to investigate the dynamics of frailty before allo-HCT. Additionally, it examines whether the association between frailty status and transplant outcomes varies depending on when frailty is evaluated, assessing the importance of its dynamic nature. Lastly, the study gathers preliminary evidence on the effectiveness of pre-habilitation programs in improving frailty before transplantation.

## Methods

### Study design, multicenter participation, and patient selection

GETH-TC is a non-profit scientific association that gathers all hospital units performing HCT in Spain and Portugal. All affiliates to the GETH-TC were invited to participate in the investigation, and fifteen institutions finally contributed to the project. All consecutive adults consulted for either autologous (auto-HCT) and allo-HCT between April 2022 and September 2023 were considered eligible for frailty evaluation after providing informed consent. Notably, the implementation of the HCT Frailty Scale has been a collaborative effort, utilizing the existing human and material resources of the HCT Units and without relying on external funding. This study was entirely observational, and the results from frailty assessments did not influence the determination of HCT eligibility or the design of the HCT process.

During the study period, 1023 patient candidates for auto- and allo-HCT have been evaluated and included in the study. This study selected the 404 allo-HCT candidates who were evaluated for frailty at first consultation and at HCT admission. Prospective data were updated in July 2024. The study was approved by the Ethics Committee of the Hospital Clínic de Barcelona and the GETH-TC, and conducted following the standards set forth by the Declaration of Helsinki.

### Frailty assessment: implementation of the HCT Frailty Scale

**Figure 1:**
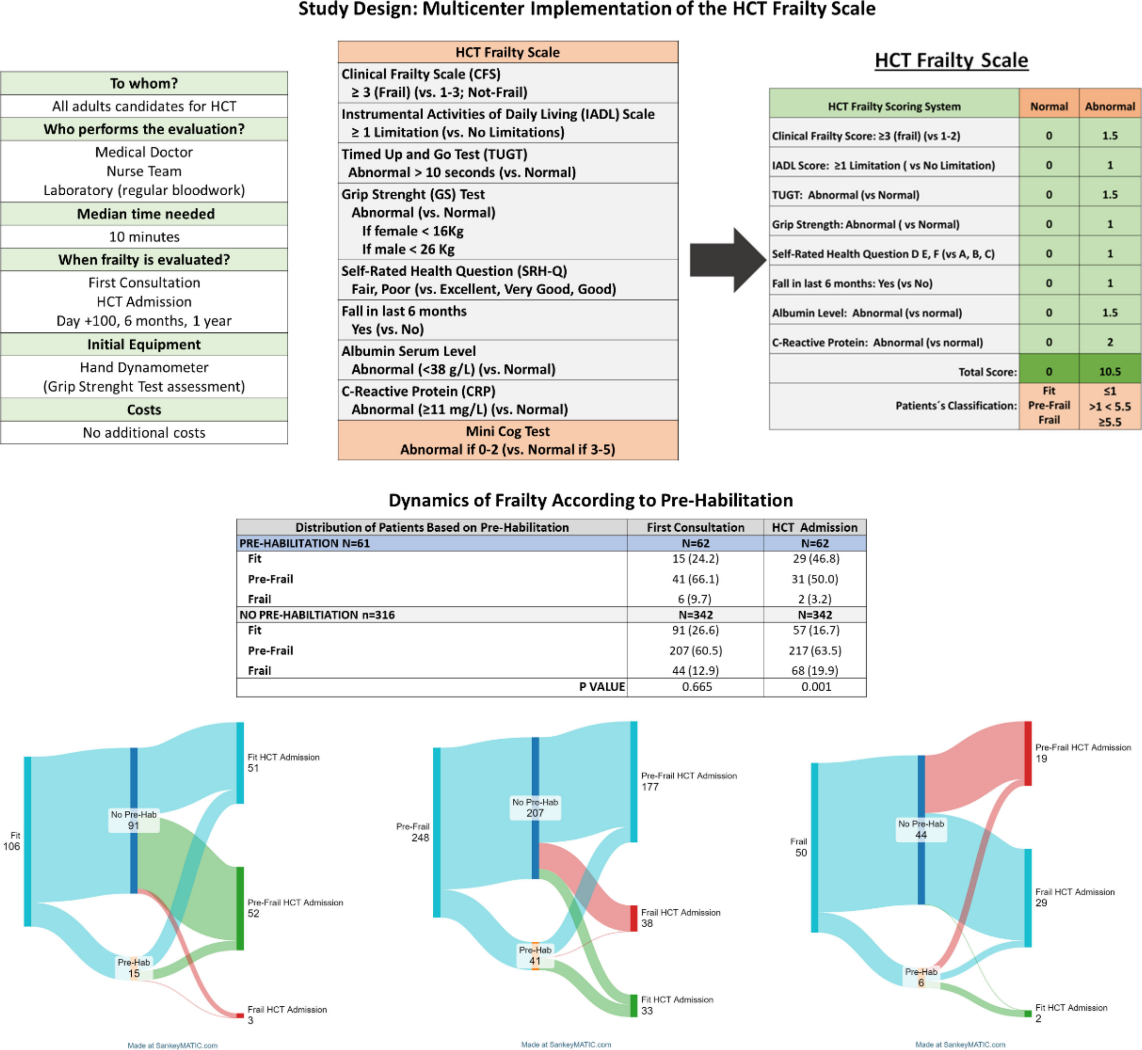
Study design and classification ability of the HCT Frailty Scale.

The research methodology commonly applied by all participating centers is outlined in **Figure 1**. The frailty syndrome was consistently assessed using the HCT Frailty Scale (26, 27), and replicating the steps followed at PMCC. To standardize the assessment process, all participants in the project underwent a remote training program led by the principal investigator with experience in the Canadian study. Candidates for allo-HCT were categorized as fit, pre-frail, and frail based on the score resulting from the sum of the weighted values of the eight variables included in the HCT Frailty Scale: Clinical Frailty Score (CFS) (28), Instrumental Activities of Daily Living (IADL) test (29), grip strength score (GS) (30), Timed Up and Go Test (TUGT) (31), Self-Rated Health question (SRH-Q), falls test, and serum albumin and C-reactive protein levels (26). Additionally, cognition was assessed using the Mini-Cog test (32).

As outlined in **Figure 1**, the study included the longitudinal evaluation of the frailty syndrome for all patients at the first HCT consultation, at HCT admission, and during post-transplant follow-up (days +100, +180 and +1-year). The frailty assessment was conducted by hematologists and nursing teams members of the HCT units participating in the study, and typically lasted between 8 to 10 minutes.

### Pre-habilitation program

In June 2022, one of the participating institutions launched a pilot pre-habilitation program for all allo-HCT candidates, regardless of age, diagnosis, or frailty status. The goal was to maintain the fitness of fit patients until HCT admission and to improve the frail or pre-frail status of those initially classified as such. The 62 adults (15.8%) consulted at this center after June 2022 were enrolled in the program. The program began within 1-7 days after the first consultation and continued until HCT admission. Pre-habilitation involved an in-person functional assessment at the Rehabilitation Service, followed by a personalized 4–8-week outpatient physical activity program monitored via telephone. To avoid delays, a dedicated clinic day was established, allowing a rehabilitation physician to schedule patients timely after their initial consultation and provide follow-up through one or two phone calls. No patient underwent in-person pre-habilitation beyond the initial assessment, and allo-HCT was never delayed to extend the program.

### Pre-transplant assessment and allo-HCT practices

The first consultation for allo-HCT was performed after stem cell donor identification, and in a median of 4 weeks before HCT admission. Each HCT institution followed internal protocols for determining patients’ eligibility for allo-HCT, donor selection and designing the HCT platform. Nevertheless, conditioning regimen intensity was generally tailored to chronological age and comorbidities. With some exceptions, patients older than 55 years with significant comorbidities received reduced intensity conditioning (RIC) regimens. Grading of acute and chronic GVHD (aGVHD and cGVHD) followed established criteria (33–35).

### Statistical analysis

The study analyzes data obtained from the HCT Frailty Scale at first consultation and at HCT admission, together with clinical and outcome information of patients included in the study. A statistical analysis chronogram is detailed in **Supplementary Material (Section 1)**. Categorical and discrete variables are presented as counts and percentages, whereas continuous variables are presented as the median and range or interquartile ranges (IQR). Univariate and multivariate regression analyses (UVA and MVA) explored the impact of frailty on outcomes overall survival (OS) and non-relapse mortality (NRM). Statistical significance was set at P<0.05. EZR software was used for statistical analysis (36).

## Results

### Frailty assessment implementation

Of the 35 HCT units members of GETH-TC dedicated to performing allo-HCT, 13 (37%) participated on the study. During the study period, 551 adults underwent allo-HCT at the participating institutions. Of these, 404 patients (73.3%) were assessed for frailty at their initial consultation and HCT admission and included in the study.

The proportion of patients evaluated for frailty, relative to the total number consulted for allo-HCT, varied across centers. In 7 programs, more than 75% of patients were evaluated, in 4 institutions, between 50-75%, and in 2 centers, fewer than 50% of patients were assessed and included in the study. These variations were largely due to differences in the availability of resources, internal coordination, and organizational practices across centers. The median time to complete the frailty assessment was 10 minutes per patient, typically performed by hematologists or nurse coordinators from each transplant team. No external funding was provided for the study, and aside from the initial material costs (hand dynamometer required for the assessment), no additional expenses were incurred during the study period.

### Frailty assessment at the first consultation

As depicted in **Table 1**, the median age of the cohort was 56 years (range: 18–76), with 95 (23.5%) patients aged 65 or older. A total of 260 (64.0%) patients were males, 151 (38.5%) had a KPS < 90%, and 60 (15.2) an HCT-CI > 3. The most frequent diagnoses were acute myeloid leukemia (33.7%) and myelodysplastic syndrome (20.8%), indicating that most patients had malignant hematological disorders.

**Table 1:** Application of the HCT Frailty Scale at first consultation in adult candidates for Allo-HCT.

Classification according to the HCT Frailty Scale evaluated at First Consultation	All Patients N = 404 (100%)	Fit N=106 (26.2)	Pre-Frail N=248 (61.4)	Frail N=50 (12.4)	P-value
Baseline Information					
Age					
Continuous (range)	56 (18-76)	51 (18-74)	58 (19-76)	54 (44-61)	0.008
Age ≥65 years	95 (23.5)	21 (19.8)	65 (26.2)	9 (17.6)	0.265
Sex					
Male	260 (64.0)	75 (70.8)	154 (62.1)	32 (62.7)	0.273
Female	144 (35.0)	31 (29.2)	94 (37.9)	19 (37.3)	
Diagnosis					
Acute Myeloid Leukemia	151 (37.0)	28 (26.4)	102 (41.1)	21 (42.0)	0.234
MDS/CMML	84 (20.8)	26 (24.5)	48 (19.3)	10 (20.0)	
Acute Lymphoblastic Leukemia	58 (14.4)	20 (18.9)	32 (12.9)	6 (12.0)	
Myeloproliferative Disorder	28 (6.9)	8 (7.5)	19 (7.7)	1 (2.0)	
Non-Hodgkin Lymphoma	48 (11.9)	14 (13.2)	26 (10.4)	8 (16.0)	
Multiple Myeloma	13 (3.2)	6 (5.7)	5 (2.0)	2 (4.0)	
Aplastic Anemia	9 (2.2)	3 (2.8)	5 (2.0)	1 (2.0)	
Others	13 (3.2)	1 (0.1)	11 (4.4)	1 (2.0)	
KPS					
<90%	151 (38.5)	26 (24.5)	100 (41.5)	25 (53.2)	0.001
Missing	12	0	9	3	
HCT-CI					
>3	60 (15.2)	9 (8.7)	43 (17.6)	8 (17.0)	0.097
Missing	8	2	4	2	
High/Very High DRI	59 (10.7)	22 (26.2)	80 (36.4)	15 (37.5)	0.630
Missing or not applicable	41	5	26	10	
Pre-Habilitation Program					
Yes	62 (15.3)	15 (14.2)	41 (16.5)	6(11.8)	0.655
Median time from first consultation to HCT					
Days (IQR)	30 (20-60)	28 (19-55)	32 (20-63)	26 (14-50)	0.175
Frailty and Functionality Evaluation: First Consultation for allo-HCT					
Clinical Frailty Score: ≥3 (pre-frail and frail)	239 (59.2)	0	189 (76.2)	50 (100)	<0.001
IADL Functional Score: ≥1 Limitation	69 (17.1)	4 (3.8)	34 (13.7)	19 (38.0)	<0.001
TUGT: Abnormal (>10 seconds)	66 (16.3)	0	34 (13.7)	32 (64.0)	<0.001
Grip Strength: Abnormal*	77 (19.1)	8 (7.5)	42 (16.9)	27 (54.0)	<0.001
Self-Rated Health Question: Fair, Poor	102 (25.2)	6 (5.7)	60 (24.2)	36 (77.0)	<0.001
Fall in Last 6 Months: Yes	52 (12.9)	13 (12.3)	17 (6.9)	22 (44.0)	<0.001
Albumin Serum Level: Abnormal (<38 g/L))	44 (10.8)	0	27 (10.9)	17 (35.0)	<0.001
C-Reactive Protein: Abnormal (≥11 mg/L)	132 (32.7)	0	102 (41.1)	30 (60.0)	<0.001
Mini-Cog Test					
Abnormal Mini-Cog test <3	33 (8.2)	6 (5.7)	17 (6.9)	10 (20.0)	0.005

*Grip strength abnormal result: <16 kg female/<26 kg male.

Allo-HCT, Allogeneic Hematopoietic Cell Transplantation; IQR, Inter Quartile Rank; IADL, Instrumental Activities of Daily Living; TUGT, Timed Up and Go Test; KPS, Karnofsky Performance Status; HCT-CI, Hematopoietic Cell Transplantation-Comorbidity Index; DRI, Disease Risk Index.

At the first consultation, 26.2% (106 patients) were classified as fit, 61.4% (248 patients) as pre-frail, and 12.4% (50 patients) as frail. As shown in **Table 1**, frail patients were older than fit patients, but had a similar median age to pre-frail patients (54 vs. 51 and 58, P=0.008). The proportions of frail patients with a KPS <90% (53.2% vs. 38.5% and 41.5%, P=0.001) and those scoring less than 3 on the Mini-Cog test (20.0% vs. 5.7% and 6.9%, P=0.005) were higher compared to fit and pre-frail patients. Additionally, frail and pre-frail patients tended to have more comorbidities, with HCT-CI > 3 present in 17.0% and 17.6% of frail and pre-frail patients, compared to 8.7% in fit patients (P=0.097).

### Pre-habilitation and frailty evaluation at HCT admission

The median time from the first consultation to HCT admission was 30 days (range, 20-50 days). At the time of HCT admission, 21.3% (86 patients) were classified as fit, 61.4% (248 patients) as pre-frail, and 17.3% (70 patients) as frail. While these proportions appeared similar to those observed at first consultation, comparisons between the two time points revealed transitions across frailty categories for some patients, regardless of their initial status and the median time from first consultation.

As illustrated in **Figure 1**, among non-pre-habilitated patients (n=342), the median of time from first consultation to HCT admission was 4 weeks (range, 3-7 weeks), and the proportion of fit patients decreased from 26.6% to 16.7%, pre-frail proportions remained similar (60.5% to 63.5%) and frail patients increased from 12.7% to 19.9%. In contrast, among the 62 (15.3%) pre-habilitated patients, the median time from first consultation to admission was 7 weeks (range, 4-8) and the improvements of the frailty state of patients were evident: the percentage of fit patients increased from 24.2% to 46.8%, while the proportion of pre-frail patients decreased from 66.1% to 50.0%, and frail patients dropped from 9.7% to 3.2% (differences statistically significant, P< 0.001).

Considering the dynamics of frailty, baseline characteristics were reassessed based on the information provided by the HCT Frailty Scale at HCT admission. As shown in **Table 2**, patient characteristics and allo-HCT procedures were similar across frailty categories, except for higher percentage of patients in the frail and pre-frail groups receiving RIC regimens (64.3% and 54.4% vs. 43.1% in fit patients, P=0.028), likely due to the higher age distribution in these groups.

**Table 2:** Impact of frailty in HCT results: information based on frailty at HCT admission.

Classification according to the HCT Frailty Scale at Admission	Fit N=86 (21.3)	Pre-Frail N=248 (61.4)	Frail N=70 (17.3)	P-value
Age				
Continuous (IQR)	48 (33-60)	58 (18-76)	58 (19-71)	0.001
Age ≥65 years	13 (15.1)	63 (25.4)	19 (27.1)	0.112
Sex				
Male	56 (65.1)	162 (65.3)	42 (60.0)	0.704
Female	30 (34.9)	86 (34.7)	28 (40.0)	
Diagnosis				
Acute Myeloid Leukemia	28 (32.6)	96 (38.7)	27 (38.6)	0.102
MDS/CMML	14 (16.3)	59 (23.8)	11 (15.8)	
Acute Lymphoblastic Leukemia	19 (22.1)	32 (12.9)	7 (10.0)	
Myeloproliferative Disorder	5 (5.8)	19 (7.7)	4 (5.7)	
Non-Hodgkin Lymphoma	12 (13.9)	26 (10.5)	10 (14.3)	
Multiple Myeloma	5 (5.9)	4 (1.6)	4 (5.7)	
Aplastic Anemia	0	7 (2.8)	2 (2.9)	
Others	3 (3.4)	15 (6.0)	5 (7.1)	
KPS <90%	18 (20.9)	93 (38.8)	40 (60.6)	<0.001
Missing	0	8	4	
HCT-CI >3	7 (8.1)	37 (15.3)	16 (23.5)	0.030
Missing	0	6	2	
High/Very High DRI	22 (26.2)	80 (36.4)	23 (39.0)	0.180
Missing or not applicable	2	28	11	
Pre-Habilitation Program (Yes)	29 (33.7)	31 (12.5)	2 (2.9)	<0.001
Days From First Consultation to Allo-HCT	48 (33-60)	30 (19-58)	25 (16-41)	0.009
Conditioning Regimen				
RIC	37 (43.0)	135 (54.4)	45 (64.3)	0.028
GVHD Prophylaxis				
PTCY-based	61 (70.1)	158 (63.7)	41 (58.6)	0.261
Donor Type				
MSD	27 (31.4)	75 (30.2)	25 (35.7)	0.214
10/10 MUD	22 (25.6)	86 (34.7)	18 (25.7)	
9/10 MMUD	17 (19.8)	26 (10.5)	7 (10.7)	
Haploidentical	20 (23.3)	61 (24.6)	20 (28.6)	
Peripheral Blood Stem Cell Grafts	83 (96.5)	240 (96.8)	66 (94-3)	0.618
Median Follow-Up (days)	470 (320-616)	391 (240-545)	306 (138-508)	0.036

IQR, Inter Quartile Rank; IADL, Instrumental Activities of Daily Living; KPS, Karnofsky Performance Status; HCT-CI, Hematopoietic Cell Transplantation-Comorbidity Index; DRI, Disease Risk Index; MAC, Myeloablative; RIC, Reduced Intensity; PTCY, Post-transplant cyclophosphamide; MSD, Matched sibling donor; MUD, Matched unrelated donor; MMUD, Mismatched unrelated donor.

Considering the transition of patients between frail states from first consultation to HCT admission, baseline characteristics and allo-HCT information of patients were reexamined based on data provided by the HCT Frailty Scale at HCT admission.

### Frailty at HCT admission and allo-HCT results

As described in **Table 3**, frail and pre-frail patients had longer median hospital stays compared to fit patients (27 and 25 days vs. 22 days, P=0.034), though no significant differences were observed in 6-month readmission rates (24.3%, 29.3%, and 28.1%, P=0.493). The cumulative incidence of aGVHD and moderate/severe cGVHD were comparable across frailty groups. For frail patients, these rates were 24.3% (P=0.236) for day +100 grade II-IV aGVHD, 8.6% (P=0.746) for day +100 grade III-IV aGVHD, and 13.0% (P=0.845) for 1-year moderate/severe cGVHD.

**Table 3:** QoL and allo-HCT results according to frailty at HCT admission.

	Fit N=86 (21.3)	Pre-Frail N=248 (61.4)	Frail N=70 (17.3)	P-value
Median duration HCT Hospitalization				
Median days (IQR)	22 (18-28)	25 (21-34)	27 (22-31)	0.034
Readmission Rate (first 180 days)	29 (32.0	75 (31.4)	16 (26.2)	0.704
Missing information	5	22	9	
Cumulative incidence of readmission at 180 days	28.1 (19.0-37.9)	29.3 (23.8-35.1)	24.3 (14.9-32.9)	0.493
Cumulative Incidence of GVHD: % (95% CI)				
Day 100 Grade II-IV aGVHD	20.9 (13.0-30.1)	26.2 (20.9-31.8)	24.3 (14.9-34.9)	0.236
Day 100 Grade III-IV aGVHD	7.0 (2.8-13.7)	8.1 (5.1-11.9)	8.6 (3.5-16.6)	0.746
1-Year Mod/Severe cGVHD	11.6 (5.7-20.0)	12.0 (8.0-16.7)	13.0 (5.6-23.5)	0.845
Main Outcomes				
Relapse	18 (20.9)	54 (21.8)	15 (21.4)	0.986
Death	19 (22.1)	74 (29.8)	31 (44.3)	0.010
Transplant-Related Mortality	20 (11.6)	43(17.3)	20 (28.6)	
Overall Survival				
1-year % (95% CI)	86.5 (76.8-92.3)	70.7 (64.3-76.1)	61.4 (48.5-71.9)	<0.001
Non-Relapse Mortality				
1-year % (95% CI)	9.8 (4.5-17.3)	17.3 (12.8-22.4)	24.4 (15.0-35.0)	0.009
Cumulative Incidence of Relapse				
1-year % (95% CI)	18.9 (11.0-28.0)	19.4 (14.6-24.6)	20.6 (11.7-30.6)	0.916

QoL of patients at the HCT Admission	Fit N=37	Pre-Frail N=109	Frail N=44	P-value
Mobility				
1	37 (100)	99 (90.8)	18 (69.2)	<0.001
2	0	10 (9.8)	4 (30.8)	
3	0	0	0	
Self-Care				
1	37 (100)	102 (93.6)	21 (80.8)	0.034
2	0	6 (5.5)	5 (19.2)	
3	0	1 (0.9)	0	
Daily Activities				
1	32 (88.9)	88 (80.0)	10 (38.5)	<0.001
2	4 (11.1)	22 (20.0)	14 (53.0)	
3	0	0	2 (7.7)	
Pain				
1	30 (83.3)	70 (64.2)	11 (42.3)	0.021
2	5 (13.9)	36 (33.0)	14 (53.8)	
3	1 (2.8)	3 (2.8)	1 (3.8)	
Anxiety				
1	32 (86.5)	59 (53.6)	12 (46.2)	0.001
2	5 (13.5)	49 (44.5)	12 (46.2)	
3	0	2 (1.8)	2 (7.7)	
% Reported	80 (70-90)	70 (60-80)	60 (35-65)	0.002

Relapse rates at 1-year did not differ significantly by frailty status, with incidences of 18.9% (95% CI: 11.0–28.0) for fit patients, 19.4% (95% CI: 14.6–24.6) for pre-frail patients, and 20.6% (95% CI: 11.7–30.6) for frail patients (P=0.009).

Lastly, quality of life (QoL) was assessed at HCT admission in 190 patients using the EQ-5D-3L test. Fit patients reported better QoL scores compared to pre-frail and frail patients, with auto-reported rates of 80%, 70%, and 60%, respectively (P=0.002).

### Frailty syndrome and transplant outcomes

**Figure 2:**
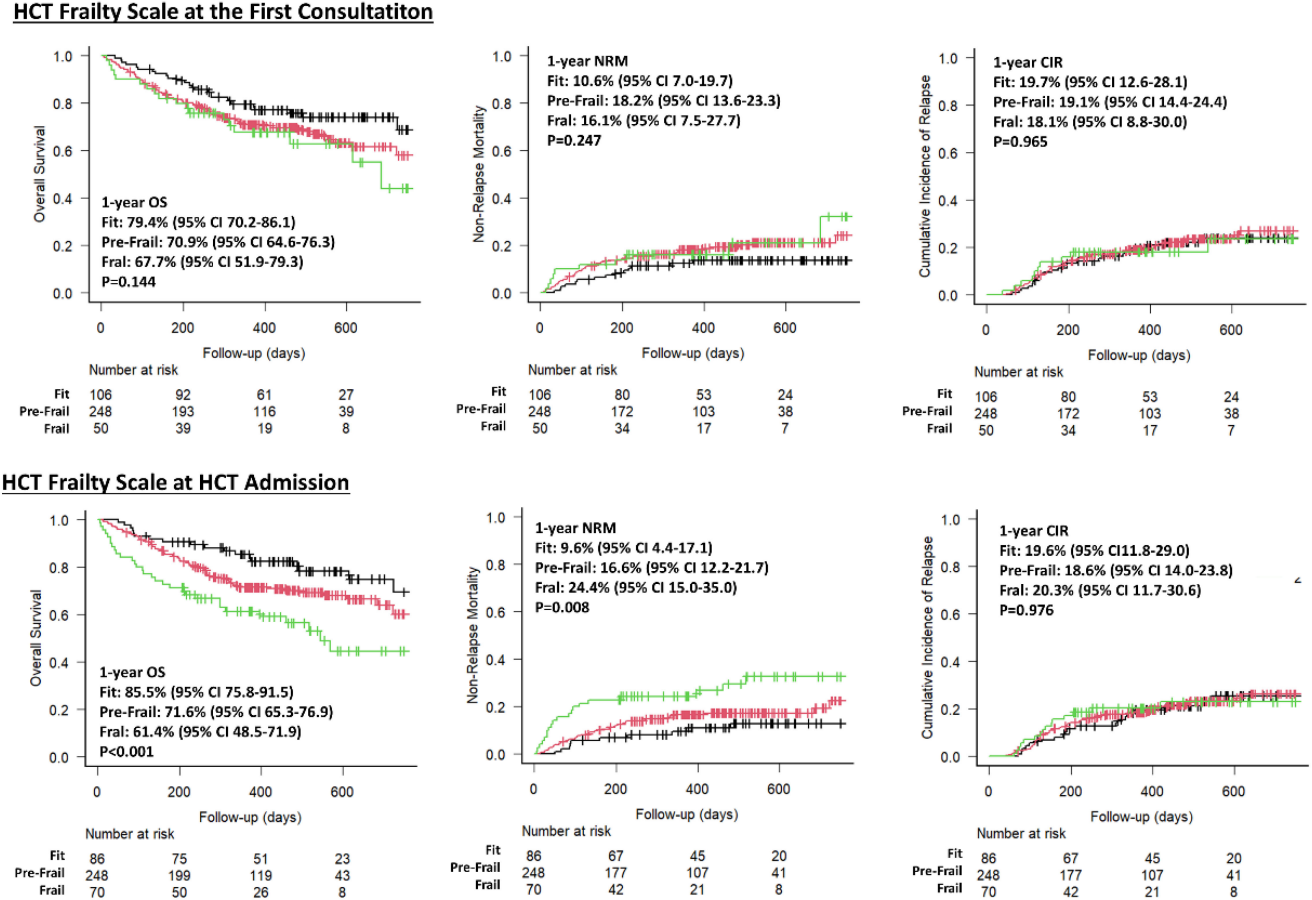
Outcomes of fit, pre-frail and frail adults undergoing allo-HCT.

With a median follow-up of 13.1 months (IQR: 7.9–18.4), 89 patients (23.2%) died, with infections and relapse being the most common causes of death. As shown in **Figure 2**, the estimated 1-year OS rates based on the HCT Frailty Scale at the first consultation were 78.4% (95% CI: 70.2–86.1) for fit patients, 70.6% (95% CI: 64.2–76.1) for pre-frail patients, and 66.4% (95% CI: 50.8–78.1) for frail patients (P=0.105). In contrast, at HCT admission, the estimated 1-year OS rates were 86.5% (95% CI: 76.8–92.3) for fit patients, 70.7% (95% CI: 64.3–76.1) for pre-frail patients, and 61.4% (95% CI: 48.5–71.9) for frail patients (P<0.001).

The effect of frailty on OS and NRM was analyzed using regression analyses. **Table 4** shows that in the UVA, the hazard ratios (HR) for OS in frail versus fit patients were 1.73 (P=0.071) at the first consultation and 2.78 (P<0.001) at HCT admission, indicating that the impact of frailty on OS differed depending on the timing of the assessment. Additionally, a trend toward worse outcomes was seen in pre-frail patients compared to fit patients at HCT admission (HR 1.56, P=0.081). Although pre-habilitation influenced frailty status at HCT admission, it did not significantly affect OS (HR 0.66, P=0.146).

**Table 4:** Frailty syndrome, overall survival and non-relapse mortality.

Univariate analysis	OS HR (95% CI)	P-value	NRM HR (95% CI)	P-value
Frailty Assessment at First Consultation				
Pre-Frail (vs. Fit)	1.43 (0.92-2.22)	0.106	1.59 (0.88-2.88)	0.12
Frail (vs. Fit)	1.73 (0.95-3.15)	0.071	1.69 (0.75-3.83)	0.20
Pre-Habilitation Program				
Yes	0.66 (0.38-1.15)	0.146	0.46 (0.20-1.06)	0.07
Frailty Assessment at HCT Admission				
Pre-Frail (vs. Fit)	1.56 (0.94-2.60)	0.082	1.58 (0.80-3.13)	0.180
Frail (vs. Fit)	2.780 (1.56-4.94)	<0.001	2.99 (1.40-6.39)	0.0046

Multivariate Analysis	OS HR (95% CI)	P value	NRM HR (95% CI)	P-value
HCT Frailty Scale (HCT Admin)				
Pre-Frail (vs. Fit)	1.47 (0.85-2.53)	0.162	1.54 (0.76-3.11)	0.220
Frail (vs. Fit)	2.30 (1.20-4.40)	0.011	2.69 (1.22-5.91)	0.013
Age				
Age ≥65 years (vs. ≤ 64 years)	1.28 (0.81-2.04)	0.282	1.59 (0.92-2.73)	0.092
KPS				
<90% (vs. 90-100%)	1.28 (0.87-1.90)	0.203	1.38 (0.86-2.21)	0.180
HCT-CI				
>3 (vs. 0-3)	0.93 (0.56-1.55)	0.801	1.07 (0.57-2.02)	0.820
DRI				
High/Very-High (vs. Low/Intermediate	1.61 (1.10-2.36)	0.013	N/A	
Conditioning Regimen				
RIC (vs. MAC)	0.93 (0.56-1.55)	0.801	1.07 (0.62-1.83)	0.800
Donor type				
HLA mismatched (vs.10/10 HLA-Matched)	1.52 (1.03-2.24)	0.031	1.82 (1.13-2.94)	0.013

MVA included variables considered clinically relevant for the HCT success. The variable pre-habilitation was not included as the results obtained from the HCT Frailty Scale at HCT admission already includes the effect of pre-habilitation on the frailty state of patients. DRI classification: Patients with non-malignant diseases were classified as Low/Intermediate Risk, and patients with CMML were evaluated according to the codification guidelines established for MDS patients. Univariate analysis of variables included in the model is described in **Supplementary Material Section 2**.

N/A, not assessed.

Differences in NRM across frailty categories were also examined (**Figure 2**, **Table 4**). Similar to OS, reclassification of patients based on frailty status following pre-habilitation altered the NRM likelihood at HCT admission compared to the initial consultation. However, at HCT admission, frail patients had a significantly higher risk of NRM than fit patients (HR 2.99, P=0.004).

The MVA including variables considered clinically relevant as determinants of transplant outcomes gave similar results than the univariate one in terms of shorter OS (HR 2.30, P=0.011) and higher NRM(HR 2.69, P=0.013) in frail patients compared to fit ones. Pre-frail patients showed a non-significant trend toward shorter OS (HR 1.47, P=0.162). In addition, patients with High/Very-High DRI/HR 1.61, P=0.013) and undergoing allo-HCT from HLA-mismatched donors (HR 1.52, P=0.031) had a shorter OS than the rest. In contrast, age over 64, KPS < 90%, and an HCT-CI > 3 were not significant predictors of OS or NRM.

### Pre-habilitation and transplant outcomes

The differences in the impact of frailty on outcomes, depending on when frailty was assessed, were attributed to the redistribution of pre-habilitated patients across frailty categories from first consultation to the time of HCT admission. To strengthen the findings, further analysis comparing pre-habilitated and non-pre-habilitated patients was conducted.

**Figure 3:**
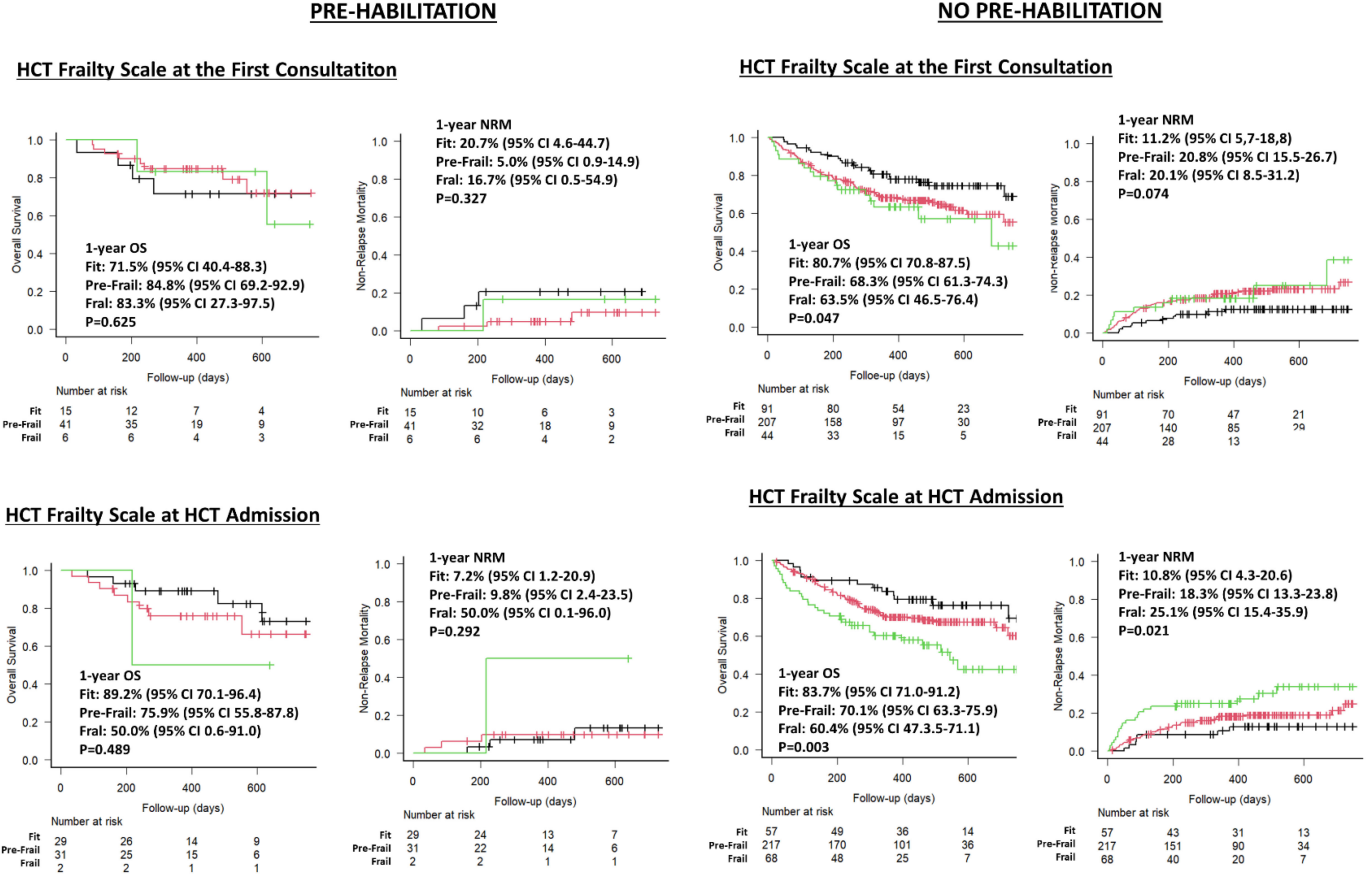
Impact of frailty and pre-Habilitation on HCT Frailty Scale classification and outcomes.

The baseline and allo-HCT characteristics were similar between both groups, except for median age (median age: 60 vs. 55 years, P=0.002) which was higher in the pre-habilitated group (**Supplementary Table 2**). As illustrated in **Figure 3**, frail patients at HCT admission had worse outcomes than fit and pre-frail ones irrespective of participation in the pre-habilitation program. However, in non-pre-habilitated patients, the differences in estimated OS rates across frailty categories were consistent whether frailty was assessed at the first consultation or at HCT admission. In contrast, for pre-habilitated patients, significant differences in transplant outcomes across frailty categories were observed only when frailty was assessed at admission. This suggests that the pre-habilitation program successfully improved patients’ fitness for transplant and that the improvement affected positively transplant outcomes.

## Discussion

This multicenter study reports the results of a frailty assessment project in the allo-HCT setting, involving 15 HCT units across Spain. It confirms that the HCT Frailty Scale, initially implemented at PMCC (26, 27), can be successfully integrated into routine clinical practice through coordinated team efforts, without requiring additional resources. Overall, 404 Spanish allo-HCT candidates were classified in fit, pre-frail, or frail categories, in proportions similar to those seen in the Canadian study. Importantly, patients’ frailty status at admission was strongly associated with transplant outcomes, with fit patients showing a higher likelihood of positive results compared to frail patients. In addition, the longitudinal evaluation of frailty documented that is dynamic and eventually reversible through individualized pre-habilitation programs.

Despite efforts to homogeneously applying the HCT Frailty Scale across the participant HCT units, there was variability across them in their engagement with the project. For example, some centers successfully evaluated over 75% of allo-HCT candidates, while others assessed fewer than 40% of potential patients. These differences mainly stemmed from the fact that not all the units had the same resources to implement the evaluation, and alert about the importance of timing coordinating efforts for the successful integration of frailty evaluations into clinical practice. This results also underscore the importance of using cost-effective tools like the HCT Frailty Scale to enable consistent application across transplant centers.

Differences were observed in the baseline characteristics of patients across various frailty levels, including comorbidities, KPS<90%, and abnormal values on the Mini-Cog test. As anticipated, given that KPS is a subjective and unidimensional measure of performance status, and considering that cognitive impairment—a key aspect of cognitive frailty—often coexists with physical frailty, the proportions of patients with KPS<90% and abnormal Mini-Cog tests were higher in frail patients (37, 38).

The analysis of the association between frailty and transplant outcomes, revealed lower likelihood of OS and higher likelihood of NRM among frail and pre-frail patients than among those classified as fit. These results did not change when including additional explanatory variables such as comorbidities, chronological age, and KPS. These results align with previously published ones on the negative impact of frailty on allo-HCT outcomes (15–25), as well as results from PMCC (26, 27). In addition, highlight the importance of implementing frailty assessments in allo-HCT practice for better patient counselling, decision making, and desiging better allo-HCT platforms.

The study further emphasizes the dynamic nature of frailty in allo-HCT candidates suggesting that frailty can potentially be reversed through pre-transplant interventions (39–42). Patients who participated in pre-habilitation showed a trend toward either improving their frailty status or maintaining their fit condition before transplantation. In contrast, some non-pre-habilitated patients experienced a decline in fitness during the pre-transplant phase. This decline was unexpected, as many patients had been discharged from prior treatments when undergo their first allo-HCT consultation.

It is presumed that consolidation therapies administered while awaiting allo-HCT, along with the cumulative toxicity of these treatments, contributed to the minimal spontaneous improvement and even worsened frailty in some non-pre-habilitated patients (**Figure 1**). Unfortunately, clinical events during this period were not collected, limiting our ability to identify specific factors influencing the negative progression of frailty in some individuals.

As shown in **Figure 3**, frailty data collected at first consultation correlated with early transplant outcomes in non-pre-habilitated patients, suggesting the transition between frailty states was not clinically significant. However, in pre-habilitated patients, frailty assessments at admission had a stronger correlation with outcomes than those at first consultation, as the variability in the frailty stages of these patients during the two time points was significantly relevant. These results highlighting the importance of tracking frailty over time and support pre-habilitation for all allo-HCT candidates to improve pre-frail and frail conditions and prevent fit patients from deteriorating (21, 43–45).

QoL at HCT admission was influenced by the patient’s frailty status. While the results in this area should be considered preliminary due to the limited number of patients evaluated, they remain significant, as QoL impairments may affect patients’ ability to cope with potential medical issues occurring during the post-transplant phase. QoL is a multifaceted concept, and the association between frailty and QoL has been minimally explored in existing studies (46).

This study primarily focused on evaluating physical frailty and QoL. However, the expansion of allo-HCT to a more diverse patients’ population together with the refinement of pre-transplant assessments underway, recommend exploring the addition of other related variables as predictors of transplant outcomes. In this respect, sarcopenia, reflecting patients’ muscle mass and strength and a key indicator of patients´ physical resilience and recovery potential, has been linked to higher likelihood of post-transplant complications and reduced OS (47, 48). Another issue that has not been considered yet in our investigation is the social dimension of frailty of transplanted patients, including economic status, caregiver availability, social support, and familial dynamics. Previous research has documented the association between patient’s ecosystem and transplant outcomes (49, 50) and more should be done in the investigation of this topic in the near future

The follow-up period in this study limited the analysis of frailty’s impact on outcomes to the first year after allo-HCT. Furthermore, while the improvement in frailty observed among pre-habilitated patients is promising, the fact that pre-habilitation was implemented at only one participating HCT unit restricts the generalizability of these findings across other institutions. Notice additionally that the information provided by the HCT Frailty Scale was not used to adjust transplant protocols or modify supportive care strategies. Future research should include longer follow-up, multicenter pre-habilitation programs, and explore how integrating frailty data into clinical decision-making and transplant planning to further improve patient outcomes.

In conclusion, this study highlights the importance of assessing frailty in clinical practice within allo-HCT settings. Frailty can be evaluated using cost-effective frailty scales such as the HCT Frailty Scale, after appropriate training and coordination (26, 27). Frailty assessment provides valuable prognostic insights into transplant outcomes, underscoring the potential benefits of pre-habilitation programs to enhance patients’ fitness before transplantation.

## Data Availability

Anonymized data would be shared only after specific request and internal consideration. Requests to access these datasets should be directed to MQS, mqsalas@clinic.cat.
