# Supplementary material for: Frailty assessment in adults undergoing allogeneic hematopoietic cell transplantation: insights from a multicenter GETH-TC study to optimize outcomes and care
Source: Front Immunol. 2025 Jan 7;15:1512154. doi: 10.3389/fimmu.2024.1512154 (PMC11747426; doi:10.3389/fimmu.2024.1512154)
Supplement: Supplementary file 1 [file DataSheet1.docx]

**SUPPLEMENTARY MATERIAL**

**SECTION 1 STATISTICAL ANALYSIS:**

The statistical analysis begins with descriptive information from the data collected through the frailty evaluation process, at first consultation and at the time of HCT admission, as well as the results of the classification of patients as fit, pre-frail, or frail according to the HCT Frailty Scale. Next, the statistical analysis moves to the evaluation of the classification of patients in the frailty scale categories as predictor of transplant outcomes. Third, the analysis combines information about the frailty classification of patients at the first consultation and HCT admission to assess the potential of pre-habilitation programs to improve the frailty state of patients and ultimately improve transplant outcomes.

**SECTION 2. Impact of Variables in HCT Outcomes: OS and NRM. Univariate Analysis**

| **Univariate Analysis** | **OS**  **HR (95% CI)** | **P-value** | **NRM**  **HR (95% CI)** | **P-value** |
| --- | --- | --- | --- | --- |
| **Age**  Continuous (IQR)  Age >64 years | 1.01 (1.01-1.03)  1.65 (1.12-2.43) | 0.012  0.010 | 1.02 (1.01- 1.04)  2.05 (1.23-3.41) | 0.094  0.003 |
| **Sex**  Female (vs. Male) | 1.07 (0.74-1.54) | 0.706 | 1.17 (0.71-1.92) | 0.52 |
| **KPS**  <80% (vs. 90-100%) | 1.57 (1.10-2.23) | 0.012 | 1.51 (0.93-2.44) | 0.091 |
| **HCT-CI**  >3 (vs. 0-3) | 1.1 (0.68-1.77) | 0.695 | 1.19 (0.64-2.22) | 0.58 |
| **Conditioning Regimen**  RIC (vs. MAC) | 1.57 (1.09-2.26) | 0.013 | 1.62 (0.99-2.65) | 0.051 |
| **GVHD Prophylaxis**  PTCY-based (vs. other) | 0.94 (0.65-1.36) | 0.772 | 1.22 (0.74-2.01) | 0.42 |
| **Donor type**  HLA mismatched (vs.10/10 HLA-Matched) | 1.55 (1.09-2.20) | 0.014 | 2.24 (1.38-3.61) | 0.001 |

**SECTION 3. Baseline Information and Frailty State of Patients According to Pre-Habilitation**

| **Frailty Assessment** | **Pre-Hab**  **N=62** | **Non-Prehab**  **N=342** | **P value** |
| --- | --- | --- | --- |
| **HCT Frailty Scale at First consultation**  **Fit**  **Pre-Frail**  **Frail** | 15 (24.2)  41 (66.1)  6 (9.7) | 91 (26.6)  207 (60.5)  44 (12.9) | 0.665 |
| **HCT Frailty Scale at Admission**  **Fit**  **Pre-Frail**  **Frail** | 29 (46.8)  31 (50.0)  2 (3.2) | 57 (16.7)  217(65.5)  68 (9.9) | <0.001 |
| **Baseline Information** |  |  |  |
| **Age**  Continuous (range)  Age ≥65 years | 60 (18-75)  18 (29.0) | 55 (18-76)  77 (22.5) | 0.002  0.026 |
| **Sex**  Male  Female | 34 (54.8)  28 (45.2) | 226 (66.1)  116 (33.9) | 0.089 |
| **Diagnosis**  Acute Myeloid Leukemia  MDS /CMML  Acute Lymphoblastic Leukemia  Myeloproliferative Disorder  Non-Hodgkin Lymphoma  Multiple Myeloma /PCL  Others | 30 (44.7)  13 (21.0)  12 (19.4)  7 (11.3)  1(1.7)  1 (1.6)  3 (4.8) | 129 (37.7)  71 (20.7)  46 (13.5)  25 (7.3)  41 (11.9)  12 (3.5)  18 (5.2) | 0.418 |
| **HCT-CI >3**  Missing | 7 (11.5)  1 | 53 (15.6)  6 | 0.389 |
| **KPS <90%**  Missing | 13 (21.0)  0 | 138 (40.4)  8 | 0.001 |
| **Median weeks from first consultation to HCT admission** | 7 weeks (4-8) | 4 weeks (3-5) | 0.001 |
| **Allo-HCT Information** |  |  |  |
| **Conditioning Regimen**  MAC  RIC | 25 (40.3)  37 (59.7) | 162 (53.6)  180 (47.4) | 0.306 |
| **GVHD Prophylaxis**  PTCY-based | 59 (95.6) | 201 (58.5) | 0.001 |
| **Donor Type**  MSD  10/10 MUD  9/10 MMUD  Haploidentical | 19 (30.6)  15 (24.2)  12 (19.4)  16 (25.8) | 108 (31.6)  111 (32.5)  38 (11.1)  85 (24.9) | 0.255 |
| **Peripheral Blood Stem Cell Grafts** | 67 (100) | 327 (95.6) | 0.188 |
| **Median Follow-up (months) (IQR)** | 392 (240-608) | 393 (236-546) | 0.890 |
|  |  |  |  |
| **Disease Relapse** | 14 (22.6) | 74 (21.6) | 0.906 |
| **Dead** | 14 (22.6) | 110 (32.2) | 0.132 |
